# Inter-Subunit Interactions across the Upper Voltage Sensing-Pore Domain Interface Contribute to the Concerted Pore Opening Transition of *K*v Channels

**DOI:** 10.1371/journal.pone.0082253

**Published:** 2013-12-10

**Authors:** Tzilhav Shem-Ad, Orr Irit, Ofer Yifrach

**Affiliations:** Department of Life Sciences and the Zlotowski Center for Neurosciences, Ben-Gurion University of the Negev, Beer Sheva, Israel; Sackler Medical School, Tel Aviv University, Israel

## Abstract

The tight electro-mechanical coupling between the voltage-sensing and pore domains of Kv channels lies at the heart of their fundamental roles in electrical signaling. Structural data have identified two voltage sensor pore inter-domain interaction surfaces, thus providing a framework to explain the molecular basis for the tight coupling of these domains. While the contribution of the intra-subunit lower domain interface to the electro-mechanical coupling that underlies channel opening is relatively well understood, the contribution of the inter-subunit upper interface to channel gating is not yet clear. Relying on energy perturbation and thermodynamic coupling analyses of tandem-dimeric *Shaker* Kv channels, we show that mutation of upper interface residues from both sides of the voltage sensor-pore domain interface stabilizes the closed channel state. These mutations, however, do not affect slow inactivation gating. We, moreover, find that upper interface residues form a network of state-dependent interactions that stabilize the open channel state. Finally, we note that the observed residue interaction network does not change during slow inactivation gating. The upper voltage sensing-pore interaction surface thus only undergoes conformational rearrangements during channel activation gating. We suggest that inter-subunit interactions across the upper domain interface mediate allosteric communication between channel subunits that contributes to the concerted nature of the late pore opening transition of Kv channels.

## Introduction

Voltage-activated potassium (Kv) channels are allosteric pore-forming membrane proteins that undergo conformational transitions between closed and open states in response to changes in electrical potential [1]–[3]. Upon opening, Kv channels allow the passive passage of potassium ions across the membrane and down their electrochemical gradient. This change in membrane permeability to K^+^ ions is fundamental for generating and shaping action potentials in nerve and muscle cells [4].

A key feature of the ability of Kv channels to mediate electrical signaling is the tight electro-mechanical coupling between their voltage-sensing and ion conduction pore domains [1]–[3], [5]. Such coupling requires that voltage-induced structural rearrangements of the voltage-sensing domains be transmitted to the pore domain so as to trigger channel opening.

**Figure 1:**
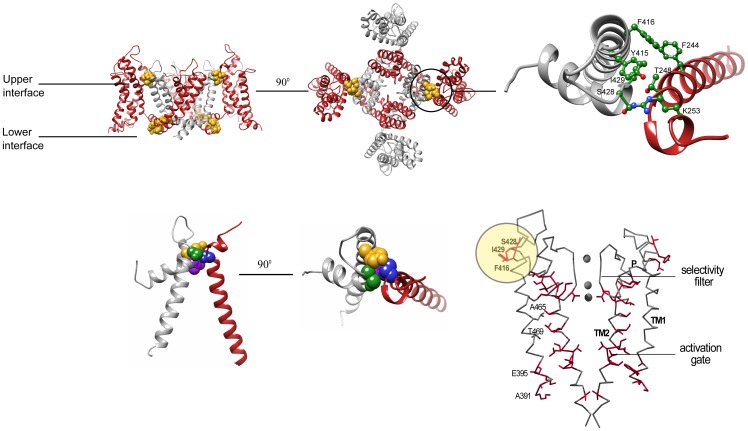
The upper inter-subunit domain interface as a potential communication hot spot contributing to the concerted pore opening transition. A(Side-view ribbon representation of the Kv 1.2 structure with the S5, P and S6 labels denoting the outer, pore and inner helices, respectively, and the S1–S4 labels denoting the trans-membrane voltage-sensor helices. For clarity and since the work here involves tandem-dimer channel proteins, the two subunits are depicted in gray and red. The primary residues involved in the lower and upper intra- and inter-subunit interaction surfaces, respectively, are depicted in a space-filling gold representation. B(Top-view ribbon representation of the Kv 1.2 structure, focusing on the upper inter-subunit interaction surface. C) Zoom-in view through the upper interface. Space-filling representations of the S1 T248, S5 Y415, P S428 and P I429 residues are highlighted in blue, gold, green and purple, respectively. D) The allosteric communication trajectory of the *Shaker* Kv channel pore domain. Gating-sensitive positions along the pore domain of the *Shaker* channel, that primarily affect the late concerted pore opening, form a connected pattern when mapped onto the closed pore conformation of the KcsA K^+^ channel. Side-chains are shown only for gating-sensitive residues (red). Highlighted in yellow circle are the gating-sensitive pore domain residues lying at the pore side of the upper voltage-sensor-pore domain interface. Adapted from [11].

Inter-domain interaction surfaces are expected to play an important role(s) in mediating such long-range coupling. However, defining the molecular details of such coupling requires atomic resolution structures of the same Kv channel in both the closed and open states. A major advance in describing the tight coupling between the voltage-sensor and pore domains at the molecular level thus came with the X-ray structure of the Kv 1.2 channel solved in the open channel state [6]–[8]. The crystal structure revealed the existence of two lower and upper inter-domain interaction surfaces (**Fig. 1A**) [8]. In the lower interface, the voltage-sensor domain of one channel subunit is coupled to the pore domain of the same subunit via the S4–S5 linker connecting the two domains in the primary sequence [7]. In contrast, the upper interaction surface corresponds to an inter-subunit interface, where voltage-sensor residues of one subunit intimately interact with pore domain residues of an adjacent subunit [7], [8] (**Fig. 1B**, and **Table S1**). In particular, the evolutionarily conserved pivotal voltage-sensor domain S1 T248 residue (*Shaker* numbering) [9], [10] is found in atomic proximity with the Y415 and S428 pore residues of a nearby subunit (**Fig. 1C**) [8], presumably forming a network of hydrogen-bonding interactions. T248 is further tightly packed against the I429 pore residue (**Fig. 1C** and **Table S1**). Interestingly, the residues lying at the pore side of the upper interface (**Fig. 1B**) were previously demonstrated to be part of a residue interaction network that contributes to the late concerted pore opening transition of the channel [11] (**Fig. 1D**). As can be seen in **Fig. 1D**, presenting the mapping of all gating-sensitive positions along the *Shaker* channel pore domain structure, in addition to the residue network that seems to connect the lower activation and upper inactivation gates, a cluster of gating-sensitive residues, including the S428, I429 and F416 residues, can be precisely mapped to the upper interface region. The Y415 residue addressed here also resides within this cluster. All other pore positions adjacent to these residues are gating-insensitive ([11], **Fig.1D**). It is, therefore, tempting to speculate that inter-subunit interactions across the upper domain interface involving the pivot T248 residue contribute to the cooperative nature of pore-opening during Kv channel gating [12].

While the lower Kv channel interaction surface undergoes extensive conformational changes, and its contribution to the electro-mechanical coupling underlying channel opening is relatively well defined [6], [13], the contribution of the upper inter-subunit interaction surface to Kv channel gating remains unclear. Evolutionary considerations, mutagenesis, covalent cross-linking and cell surface expression studies all indicate that the residues spanning the upper interaction surface are found in close spatial proximity and play an important role in Kv channel function [9]-[10], [14]. These analyses, however, §do not provide mechanistic information regarding possible structural rearrangements associated with this interface during Kv channel gating. This is due, in part, to the severe reduction in channel cell surface expression that occurs upon mutating the pivotal voltage-sensor upper interface T248 residue [9]–[10].

Hence, to determine whether the upper interaction surface undergoes structural rearrangement during both Kv channel activation and slow inactivation gating, we measured the gating sensitivity of the appropriate transition upon mutation of the upper interface residues and assessed the state-dependency of interactions across the interface. Gating sensitivity can serve as a reporter of structural rearrangements involving these residues during the transition studied [11]. Furthermore, state-dependency of such interactions is the hallmark of structural rearrangements that occur during the thermodynamic transition studied. In the present study, upper interface residue gating sensitivity and state-dependent interactions were assessed by combining electrophysiology recordings of wild type and upper interface mutants of the archetypal *Shaker*-*IR* Kv channel, introduced in the context of a tandem-dimer channel construct [15], followed by high-dimensional double-mutant cycle thermodynamic coupling analysis [16]–[18]. Our results reveal that the upper domain interface undergoes moderate structural rearrangements during activation gating but not during the subsequent slow inactivation gating transition. As such, we suggest that a primary role of inter-subunit interactions across the upper domain interface is to transmit allosteric communication between the Kv channel subunits, thus contributing to the concerted nature of the late pore-opening transition of the *Shaker* Kv channel.

## Materials and Methods

### Molecular biology

In this study, we used the *Shaker-IR* Kv channel variant lacking the N-type inactivation domain (residues 6–46) [19]. In addition, to facilitate detection of inter-subunit interactions, a tandem-dimer Kv channel gene construct was used wherein two identical wild type *Shaker* genes (designated A and B) were connected in tandem by a stretch of nucleotides encoding a flexible loop [15]. The tandem-dimer gene contains one start codon and one stop codon that are flanked by unique restriction sites at the 5′ and 3′ ends upstream and downstream of the start and stop codons, respectively. Mutations in one or two subunits were generated by standard ligation (T4 ligase) of wild type or mutated subunit fragments with a template fragment (pGEM), all cut with the appropriate restriction enzymes. Alanine substitutions were chosen, as these are the preferred reference state in DMC coupling analysis [20]. mRNA for wild type or mutant channels was synthesized using T7 RNA polymerase, as previously described [11]. Mutations were introduced by the QuickChange method (Stratagene) and confirmed by sequencing of the entire cDNA.

### Electrophysiology

Activation gating: K^+^ currents were recorded from the *Shaker*-*IR* channel under conditions of two-electrode voltage clamp (OC725B, Warner Instruments) 1–3 days after tandem-dimeric wild type or mutant *Shaker* mRNA injection, as described previously [11]. Voltage-activation curves were obtained using a standard tail current protocol and represent the average of recording from more than 20 different oocytes (See also data analysis section). Care was taken to measure only those oocytes expressing K^+^ tail currents in a narrow range of 2–5 µA. Slow inactivation gating measurements were performed in a bath solution containing (in mM): 93 NaCl, 4 KCl, 1 MgCl_2_, 0.3 CaCl_2_, 5 HEPES, pH 7.4. The onset of slow inactivation was induced by applying a prolonged, 20 sec, depolarizing pulse. Recovery from slow inactivation was measured using a modified pair-pulse protocol. In particular, the onset of inactivation was initiated using a prolonged depolarizing pre-pulse to 0 mV, immediately followed by a multi-sweep recovery protocol where the onset of test pulses was initiated at progressively increasing *Δ*t intervals. The inter-sweep time interval was set to 40 sec. The fractional recovery was calculated as the peak current level of the test pulse, relative to the peak current of the conditioning pre-pulse (both corrected for the steady-state inactivation level). Recovery from inactivation revealed two fast and slow components. Only the slow component was affected upon mutation or upon changes in external potassium concentration. As expected, increasing external [K^+^] resulted in faster recovery rates and slower onset of inactivation rates. Furthermore, the amplitude of the fast phase did not change upon increasing the onset of inactivation pulse duration, thereby excluding the possibility that this fast phase represents a non-inactivated channel population. While we do not know the source of the fast phase in our measurements, it is, however, evident that the slow component describes recovery from inactivation associated with selectivity filter rearrangement.

### Data analysis

Voltage-activation curves were fitted to a simple two-state Boltzmann equation: I/I_max_ = (1+*e* ^−ZF(V−V^_1/2_^)/RT^)^−1^, where I/I_max_ is the normalized tail current amplitude of the *Shaker* channel, Z is the activation slope factor, V_1/2_ is the half-activation voltage, and T, F and R have their usual thermodynamic meanings. Differences in V_1/2_ between wild type and mutant channels (∼10–15 mV) were assessed for statistical significance using a two-sided Student's *t* test, where *n* for each group is 20–30 oocytes. The null hypothesis (that the observed differences are due to chance) was rejected based on a *p-value* smaller than 0.001. Free energy differences between closed (**C**) and open (**O**) states of the wild type or mutant channels were parameterized based on gating shifts and slopes according to *ΔG*_o_ = (−RT*ln*([**O**]/[**C**])) = ZFV_1/2_. Time constants for the onset of inactivation were calculated by fitting the data to a single exponential equation as follows: I = I_0_+Ae^−t/τo^, where I is the measured current, I_0_ is the steady-state current level, *t* is the elapsed time, τ_o_ is the time constant for onset of inactivation, and A is an amplitude constant. The adequacy of single exponential fits was judged by residual plots, with all exhibiting random distribution of the residual current around zero. Assuming a simple inactivation gating transition, described by the open (**O)** state to the inactivated (**I**) state, values for the forward and backward inactivation rate constants (*k_f_* and *k_b_*, respectively) at 0 mV were derived using the time constant for the onset of inactivation (τ_o_ = 1/(*k_f_*+*k_b_*)) and the steady state amplitude factor (*A*_ss_ = *I*_ss_/*I*_peak_ = *k_b_*/(*k_f_*+*k_b_*), where *I*_ss_ and *I*_peak_ are the respective steady-state and peak current levels. Values for the equilibrium constant of inactivation gate closure (**O** ↔ **I** transition) *K*_I_ were obtained by dividing the forward and backward inactivation rate constants (*K*_I_ = *k_f_*/*k_b_*). Time constants for recovery from inactivation were calculated by fitting the data to a double-exponential function. Standard errors in *K*_I_ *ΔG*_open_, *ΔG*_inactivation_ and in the coupling free energies (*Δ* ^2^ *G_(i,j)_* and *Δ* ^3^ *G*_(*i,j,k*)_, as determined from double-mutant cycle analysis) were calculated by standard linear error propagation [11]. To determine the extent of residue coupling, a cut-off value of 0.5 kcal/mol was used in the DMC analysis. A visual outline of additivity in the DMC analysis (gray dashed lines in the appropriate coupling figures) was made possible by generating an extrapolated curve calculated based on the sum of changes in the midpoint activation voltage (V_1/2_) of the two single-mutants and an averaged value of their slopes (Z).

## Results

### Upper Interface Residue Mutations Stabilize the Closed Channel State

In the open Kv 1.2 channel structure, the C terminal end of the S1 helix is tilted towards the C and N termini of the respective S5 and pore helices of the adjacent subunit, forming an intimate interaction surface primarily involving the T248, Y415, S428 and I429 residues [8] (**Fig. 1C**). The uniqueness and importance of these residues to Kv channel function has been realized in previous studies, independently addressing the voltage sensor and pore sides of the upper domain interface [9]–[11]. Specifically, it has been demonstrated that the pivotal S1 T248 residue is absolutely essential for proper channel maturation and cell surface expression [10]. Bioinformatics correlated mutation analysis further revealed T248 to be a part of a co-evolved interaction surface, hinting at the importance of this residue for the mechanics of channel gating [9]. As stated above, the residues lying at the pore side of the upper interface were previously demonstrated to be a part of a residue interaction network important for the late concerted pore opening transition of the channel [11] (**Fig. 1D**). The relation of these pore network residues to the voltage sensor T248 residue has, however, yet to be addressed. With this in mind, we initially focused on the T248 residue.

**Figure 2:**
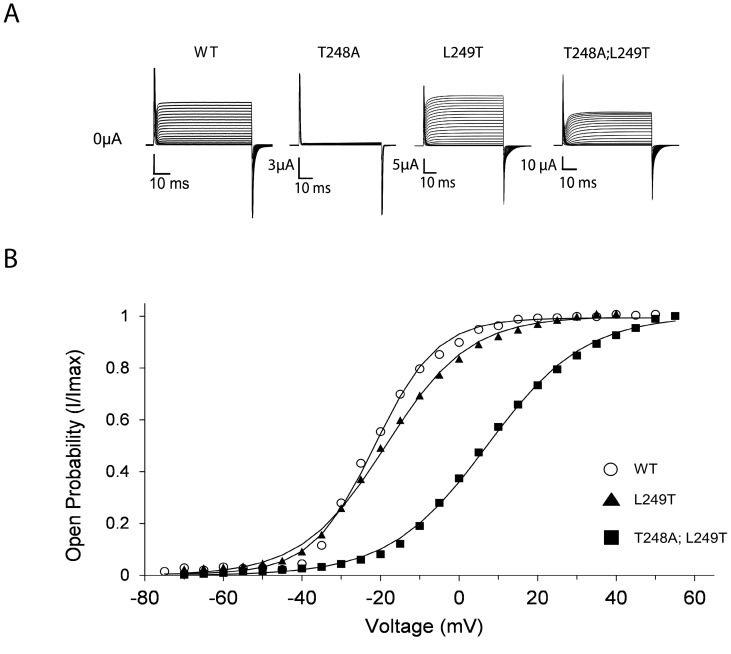
The upper interface pivotal T248 residue affects channel maturation and gating. **A**) K^+^ currents recorded from *Xenopus laevis* oocytes expressing the wild type channel, the T248A and L249T single-mutants and the corresponding T248A;L249T double-mutant. **B**) Voltage-activation data for the K^+^ channel proteins indicated in (**A**). In all panels, smooth curves correspond to a two-state Boltzmann function. For clarity, in this and in other figures, no error bars are plotted for the different data points of the different curves. These values, however, are in a range of 5% of the reported values.

As reported previously [9]–[10], mutating T248 to alanine (T248A) in all four subunits resulted in a severe reduction in K^+^ current expression, hampering evaluation of the effect of mutating this position on channel gating (**Fig. 2A**). To, therefore, separate channel gating and maturation effects, we generated the L249T mutant and the corresponding T248A;L249T double-mutant. As can be seen in **Fig. 2A**, introducing the L249T mutation either alone or on the background of the T248A mutation resulted in normal potassium current levels. The T248A;L249T double-mutant thus suppresses the non-expressing phenotype of the T248A single-mutant, further pointing to the requirement of a Threonine residue in this region of the protein for proper channel maturation. As can be seen in **Fig. 2B**, the voltage-activation curve of the T248A;L249T double-mutant is shifted to the right along the voltage axis (*ΔV*_1/2_ = 28 mV), as compared to the wild type channel. This effect on gating is primarily a result of the T248A mutation, as the L249T single-mutant exhibits a wild type-like activation curve (**Table 1**). These results imply that the pivotal T248 residue is important for both channel maturation and gating. Interfering with the intimate native interaction context of this residue, as occurs in the open Kv 1.2 channel state, affects gating and leads to stabilization of the closed channel state, as further manifested when other perturbations at the 248 position were introduced (**Fig. S1**). Considering the high conservation of the S1 T248 residue (**Fig. S1**), it is of note that a closed state-stabilizing effect is also observed upon alanine mutation of the analogous threonine in Kv 7.2 and Kv 7.3 channel family members [21].

**Table 1:** The influence of upper interface T248 and L249 mutations on activation gating of the S*haker* Kv channel^a^

Channel protein^b^	K^+^ current?	V_1/2_ (mV)	Z	*ΔG*_open_ (kcal/mol)
Wild type	Observed	−21.6±0.2	3.20±0.07	−1.64±0.04
T248A	NO	NA	NA	NA
L249T	Observed	−19.2±0.2	3.04±0.05	−1.36±0.02
T248A;L249T	Observed	6.4±0.2	1.89±0.02	0.28±0.01

**Fig. 2**.^a^ The table displays the gating parameters of all single- and double-mutants used to assess the effect of T248 and or L249 mutation on channel maturation and gating, as addressed in

^b^ Mutants were generated using a monomeric gene construct such that all four subunits carry the indicated mutation.

**Figure 3:**
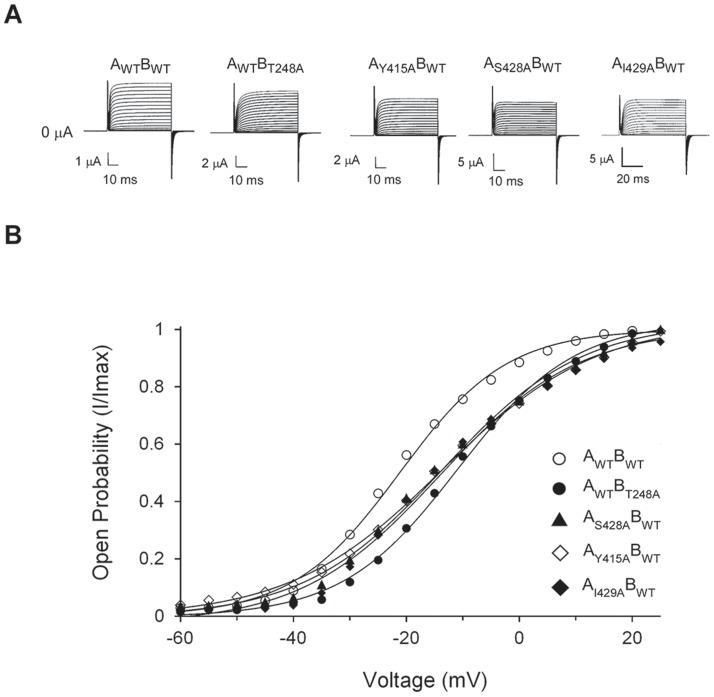
Mutations across the upper interface stabilize the closed channel state. **A**) K^+^ currents recorded from *Xenopus laevis* oocytes under two-electrode voltage clamp expressing the tandem-dimer wild type (WT) and the upper interface T248A, Y415A, S428A and I429A *Shaker* channel mutants (see **Materials and Methods**). **B**) Voltage-activation data for the tandem-dimer K^+^ channel proteins indicated in (**A**). Smooth curves correspond to a two-state Boltzmann function. The observed effects on gating are statistically significant, as judged by comparing the wild type and mutant activation midpoints using a two-tail Student's *t* test (*n_1_,n_2_*>20; *P-value* <<0.001) (see **Materials and Methods**).

Next, to quantitatively address the contribution of the upper interface residues to channel gating, we introduced alanine mutations at the T248, Y415, S428 and I429 positions in a tandem-dimer channel construct. The use of a tandem dimer channel construct is necessary for the accurate assessment of inter-subunit interactions across the upper interface and may further serve to alleviate a non-functional phenotype realized upon mutating all four subunits. In particular, we were intrigued by the phenotype of the concatenated tandem-dimer T248A channel mutant presenting two native T248 residues and two (diagonal) T248A mutations (the A_WT_B_T248A_ mutant). As reflected in **Fig. 3A**, all upper interface point mutations indeed resulted in functional tandem-dimer channels. In each tandem-dimer channel, the upper interface mutant activation curve is shifted to the right along the voltage axis, as compared with the tandem-dimer wild type channel (**Fig. 3B**), further implying a closed-state stabilization effect upon mutation. Although moderate, the observed effects on gating are statistically significant, based on a comparison of the mutant and wild type activation midpoints (*P-value* <<0.001) (See **Materials and Methods**). Furthermore, the results for the S428A and I429A mutants are coherent with previous results [11]. Considering that pore network residues, were found to primarily affect the late pore opening transition [11], we used Boltzmann's activation midpoint (V_1/2_) and slope (Z) to quantitatively assess the effects of mutation on the energetics of channel opening. This energy estimate for channel gating is possible as, for these residues, a linear correlation was found to exist between Boltzmann energies and actual energies derived based on the more realistic 16-state gating scheme ([11] see Fig. 3A therein). As can be seen in **Table 2**, a closed state stabilization effect of similar magnitude (∼1 kcal/mol) was calculated for the different mutants. The observed gating effect can be rationalized based on the Kv 1.2 structure solved in the open channel state [8] as breaking direct interactions that stabilize the open state should lead to a net stabilization of the closed channel state, as was indeed observed. Whether these residues also affect early voltage sensor transitions is unknown, since the reduction in channel cell surface expression upon mutating the indicated residues (in particular, the T248A mutant) hampers gating current *Q-V* measurements. This further prevents the obtaining of more accurate estimates for the overall channel gating free energies [22].

**Table 2:** The influence of upper interface single-, double- and triple-mutations on activation gating of the tandem-dimer *Shaker* Kv channel^a^

Channel protein^b^	V_1/2_ (mV)	Z	*ΔG*_open_ (kcal/mol)
Wild type (A_WT_B_WT_)	−23.7±0.2	3.10±0.12	−1.70±0.06
T248A (A_WT_B_T248A_)	−10.3±0.7	2.32±0.15	−0.55±0.05
Y415A (A_Y415A_B_WT_)	−13.3±0.3	2.04±0.05	−0.63±0.02
S428A (A_S428A_B_WT_)	−14.5±0.8	2.18±0.10	−0.73±0.05
I429A (A_I429A_B_WT_)	−11.0±0.1	2.55±0.08	−0.65±0.02
Y415A, T248A (A_Y415A_B_T248A_)	−11.2±0.1	2.42±0.05	−0.62±0.01
S428A, T248A (A_S428A_B_T248A_)	−10.8±0.3	2.43±0.15	−0.60±0.04
I429A, T248A (A_I429A_B_T248A_)	−6.7±0.3	1.86±0.10	−0.29±0.02
Y415A,S428A (A_Y415A;S428A_B_WT_)	−13.8±0.3	2.43±0.08	−0.77±0.03
Y415A,S428A,T248A (A_Y415A;S428A_B_T248A_)	−10.3±0.4	2.19±0.08	−0.52±0.03

**Figure 4:**
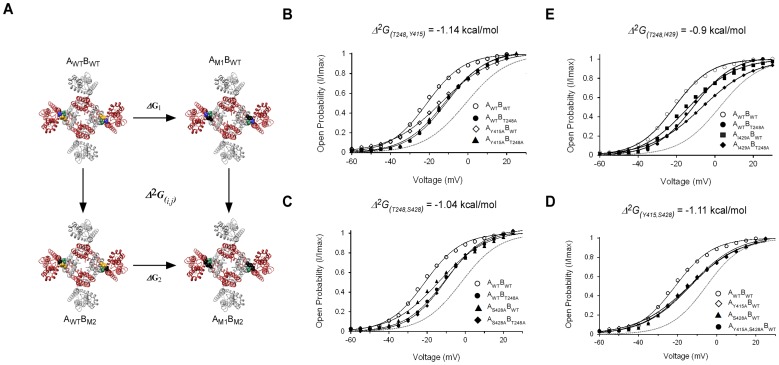
Residues across the upper interface are thermodynamically coupled in a state-dependent manner. **A**) Thermodynamic double-mutant cycle coupling analysis of voltage-dependent gating applied to inter-subunit interactions across the upper interface. The equilibrium between the closed and open states of the tandem-dimer wild type (A_WT_B_WT_), single-mutant (A_M1_B_WT_ or A_WT_B_M2_), and double-mutant (A_M1_B_M2_) channels is related by a thermodynamic square that enables measurement of the magnitude (*Δ* ^2^ *G_(i,j)_*) and state-dependency (sign of coupling, whether ‘+’ or ‘−’) of pairwise coupling between residues *i* and *j* (positions 1 and 2) of the identical A and B subunits, respectively. In the thermodynamic cycle shown, the interaction between the Y415 and T248 residue pair is considered. Mutations (to alanine) are represented by the black color. **B–E**), Voltage-activation curves for the four channel proteins comprising the double-mutant cycle measuring the coupling free energy between the upper interface T248 and Y415 (**B**), T248 and S428 (**C**), T248 and I429 (**D**) and Y415 and S428 (**E**) residue pairs. Smooth curves correspond to a two-state Boltzmann function. The smooth gray curve in each panel corresponds to an extrapolated voltage-activation curve that assumes additivity of the effects of the two single-mutations on gating (see **Materials and Methods**).

**Figure 5:**
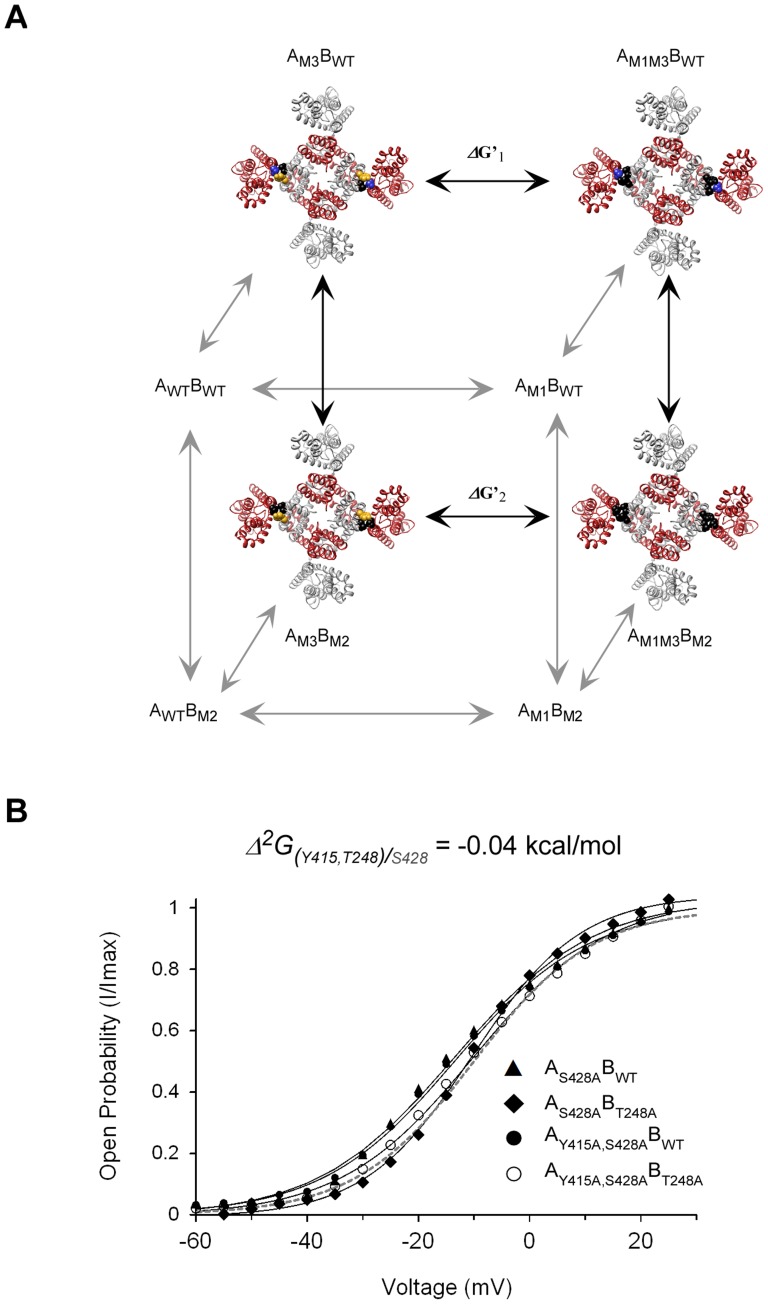
The upper interface T248, Y415 and S428 residue triad forms a network of coupled interactions during activation gating. A) A thermodynamic cubic construct was used to measure mutual three-dimensional coupling (*Δ* ^3^ *G_(i,j,k)_*) between the three upper interface residues. *Δ* ^3^ *G* is calculated by subtracting the coupling free energy between any residue pair in the presence and absence of the native third residue (front and back faces of the cube, respectively). For clarity, only the channel proteins of the back face are explicitly represented. The front face proteins are described in the legend to Fig. 4A. B) Voltage-activation curves for four channel proteins comprising the thermodynamic double-mutant cycle measuring the coupling free energy between the upper interface T248 and Y415 (*i,j*) residues on the background of the mutated S428 residue (*k*). The same cycle, only in the presence of the native third position, is considered in Fig. 4B (front face of the cubic construct shown in (A)).

**Figs. 4** and **5**.^a^ The table displays the gating parameters of all single-, double- and triple-mutants used to calculate the coupling free energies presented in

^b^ Mutants were generated using the tandem-dimer gene construct. Any mutation is thus present in only two diagonal channel subunits.

### Upper Interface Residues Form a Network of Coupled State-Dependent Interactions

The above results point to the involvement of interactions across the upper interface in activation gating. To evaluate the contribution of such putative interactions to channel gating, we employed high-dimensional double mutant cycle (DMC) coupling analysis [16], [18]. Such analysis enables one to evaluate the strength of interaction(s) between two or more residues *Δ^n^G* (where *n* indicates the number of coupled residues) and their state-dependency, *i.e.*, whether the interaction is stronger in one state relative to the other. This property of DMC analysis is reflected in the plus (+) or minus (-) sign descriptor of the coupling free energy. In the case of the closed to open channel equilibrium studied here, the magnitude (*Δ* ^2^ *G*_(*i,j*)_) and sign of the coupling free energy of the three upper interface residue pairs (*i,j*) can be evaluated by measuring the activation curves of four tandem-dimer channel proteins, namely the wild type protein, the two single-mutant proteins, and the corresponding double-mutant [11], [17]. **Fig. 4A** presents such an analysis for the interaction between the S1 T248 and S5 Y415 residue pair across the upper interface. As can be seen, only two diagonal upper interfaces are perturbed in the DMC analysis of tandem-dimer proteins. The results of DMC analysis of four upper interface residue pairs are presented in **Figs. 4B–E**, with the V_1/2_ and Z gating parameters and the channel opening free energies (*ΔG*_o_) for each single- and double-mutant channel listed in **Table 2**. As is qualitatively evident, all four residue pairs show a non-additive effect on channel gating. This can be intuitively realized by noting that the midpoint activation voltage of the double-mutant channel protein deviates from the midpoint activation voltage of a curve (dashed gray line) that assumes an additive effect of the two single-mutations (see **Materials and Methods**). This typical non-additive behavior suggests that the four upper interface residue pairs are coupled energetically. When parameterized, based on gating shifts and slopes, coupling free energy values (*Δ* ^2^ *G*_(*i*,*j*)_) in the range of −1 kcal/mol were calculated for the different (*i*,*j*) residue pairs (**Table 3**, second column from left). Indeed, the energetic coupling among the residue pairs is not dramatic. These effects, however, are significant, since when the T248 voltage-sensing domain residue is tested for coupling with the Y415 pore residue of the same subunit, no coupling was detected (a coupling value in the range of 0.2 kcal is obtained; value in grey in **Table 3**). The results lead to two conclusions. First, the spatial proximity of the residues tested across the upper inter-subunit interface, as indicated by the Kv 1.2 open channel structure ([8], **Fig. 1C**) and subsequently detected by cross-linking analysis [9], is manifested here in functional terms. We find that all proximal residue pairs at the upper interface are coupled energetically. Second, coupling free energies calculated between any of the residue pairs yield a minus (‘-’) coupling sign, indicating the coupling to be stronger in the open channel state than in the closed state by ∼1 kcal/mol, consistent with the Kv 1.2 open channel structure [8].

**Table 3:** 2^nd^- and 3^rd^-order coupling free energies between the upper interface residues of the *Shaker* Kv channel during activation gating.

*Δ^3^G_(i,j)k_* (kcal/mol)^b^	*Δ^2^G _(i,j)/k_* (kcal/mol) mutated *k*	*Δ^2^G_(i,j)_* (kcal/mol) native *k*	*(i,j)k* residue triad^a^
−1.10±0.20	−0.04±0.08	−1.14±0.10 (**0.2**±**0.05**)c	(T248,Y415) S428
−1.10±0.20	−0.01±0.05	−1.11±0.11	(Y415,S428) T248
−1.10±0.20	0.06±0.07	−1.04±0.09	(T248,S428) Y415
ND	ND	−0.90±0.09	(T248,I429) Y415

*i,j*) residue pairs, as indicated in **Figs**. **4B–E**, was measured in the presence (*Δ^2^G_(i,j)_*) or absence (*Δ2G _(i,j)/k_*) of the native third *k* position.^a^ The coupling free energy of the (

*k* on the *(i,j)* interaction pair is (*Δ^3^G_(i,j)k_*), calculated by subtracting the free energies of coupling in the presence or absence of the native third *k* position. Considering the symmetry of the three-dimensional mutant construct (see **Fig. 5**), any of the three residue combinations yields a similar value for *Δ^3^G_(i,j)k_*.^b^ The effect of residue

*Δ^2^G_(i,j)_* coupling value between the indicated pair where both T248 and Y415 residues are in the same subunit.^c^ Shown in parenthesis is the

Since a network is defined by at least three members, we next focused on the intimately packed T248, S428 and Y415 residue triad and measured the mutual 3^rd^-order coupling (*Δ* ^3^ *G*_(*i,j*)*k*_) among these residues [16], [18]. The thermodynamic cubic construct presented in **Fig. 5A** demonstrates how a three-way residue coupling that evaluates the effect of a third residue, *k* (*e.g.* S428), on the magnitude of coupling between a *i,j* residue pair (T248 and Y415, in this case), can be measured. The impact of residue *k* is obtained by subtracting the pairwise coupling free energy between *i* and *j* in the presence and absence of the native residue *k*, as given by the front and back faces of the cube, respectively. **Fig. 5B** presents the activation curves of the four proteins needed for estimating the coupling free energy between T248 and Y415 on the background of the S428A mutant. Coupling between the T248,Y415 residue pair is abolished upon mutation of the S428 position (*Δ* ^2^ *G*_(*i,j*)*/*_k = ∼0.1 kcal/mol; compare with **Fig. 4B**). The same is true for the other two residue pairs, where coupling is dramatically reduced upon mutating the third position (**Table 3**, compare the two middle columns). A three-way coupling value of −1.10 (±0.20) kcal/mol is calculated for the residue triad, indicating that coupling between any residue pair is absolutely dependent on the presence of the third native position. Our results are thus consistent with the existence of a network of mutually interacting residues along the upper interface that contribute to activation gate opening.

### Upper Interface Residues are Gating-Insensitive and Reveal No State-Dependent Interactions During Slow Inactivation Gating

**Figure 6:**
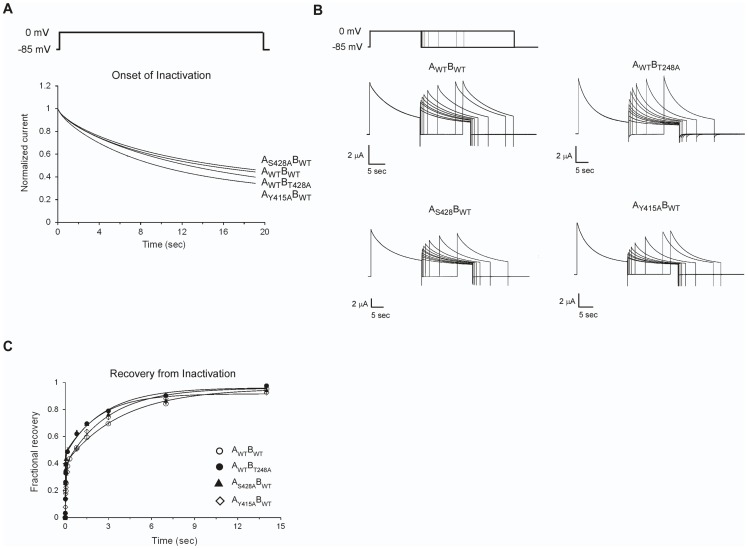
Upper interface mutations do not affect slow inactivation gating. **A**) A typical command protocol, along with the potassium currents elicited from the wild type and upper interface T248A, S428A and Y415A mutants during the onset of slow inactivation (normalized relative to the peak current amplitude). **B**) A typical command protocol, along with the elicited potassium currents for the wild type and upper interface T248A, S428A and Y415A mutants during the onset of and recovery from slow inactivation, is depicted. **C**) Normalized kinetics for the recovery from inactivation of the different mutants indicated in (**B**). The data were fitted to a double-exponential function. The time constants of the slow phase (indicative of recovery from inactivation (see **Materials and Methods**)) for the T248A, Y415A and S428A mutants are reported in **Table 4**.

Next, to address whether the upper inter-subunit domain interaction network also plays a role during the later slow inactivation gating transition [23−26], we applied high-dimensional DMC formalism to channel inactivation gating by measuring the kinetics of onset of slow inactivation (see **Materials and Methods**) for all eight upper interface channel proteins at the corners of the thermodynamic cubic construct described in **Fig. 5A**. A command protocol, along with the potassium currents elicited from the wild type and different upper interface single-mutants during the onset of inactivation, is presented in **Fig. 6A**. As can be seen, minor changes in current development over time are observed for all upper interface single-mutants. The data for the single-mutants and for all other double- and triple-mutants were fitted to a single-exponential function, with the values for the time constants for the onset of inactivation (τ_o_) and for the steady-state inactivation amplitude factor (*A*_ss_) reported in **Table 4**. A minor, less than a two-fold change is observed for τ_o_ upon upper interface single-mutation. Such changes are much less than that reported for mutations of the S5 E418 position, another proximal residue that is not part of the upper interface [27]. In this latter study, a 200-fold change was observed for τ_o_ upon cysteine substitution, an effect that could be rationalized by a breaking of the interactions of E418 with its adjacent S6 residue counterparts [27]. Assuming a simple inactivation gating transition, described by the open (**O**) to inactivated (**I**) states, values for the forward and backward slow inactivation rate constants (*k_f_* and *k_b_*, respectively) were derived using the time constant for the onset of inactivation and the steady state amplitude factor (according to *τ*_o_ = 1/(*k_f_*+*k_b_*) and *A*_ss_ = *k_b_*/(*k_f_*+*k_b_*); see **Materials and Methods**), and are listed in **Table 4**. As can be seen, the gating insensitivity of the upper interface single-mutants during inactivation gating, qualitatively realized in **Fig. 6A**, is further reflected in the forward and backward inactivation time constants and in the equilibrium constant for inactivation gate closure, *K*_I_ ( = *k_f_*/*k_b_*) (**Table 4**). Measurements of recovery from inactivation for all single-mutants, using a pair-pulse protocol (**Fig. 6B**), further revealed minor changes in the kinetics of recovery, as compared to the wild type channel (**Fig. 6C**). The data were fitted to a double-exponential function yielding estimates for the values of the fast and slow phases. Only the slow phase was sensitive to mutation and to changes in external potassium concentration and is thus indicative of the recovery from inactivation associated with selectivity filter rearrangements (See also **Materials and Methods**). Inspection of the recovery time constants of the different upper interface single-mutants, reported in **Table 4** (rightmost column), indeed reveals minor changes upon mutation, consistent with the entry to inactivation data.

**Table 4:** The influence of upper interface single-, double- and triple-mutations on slow inactivation gating of the tandem dimer *Shaker* Kv channel^a^

Channel protein	Onset of inactivation time constant (τ_o_) (msec)	Steady-state inactivation amplitude (A_ss_)	Forwards inactivation time constant (τ_f_) (msec)	Backwards inactivation time constant (τ_r_) (msec)	Equilibrium constant for inactivation gate closure (*K*_I_)^b^	*ΔG*_inactivation_ c (kcal/mol)	*ΔΔG*_inactivation_ e (kcal/mol)	Recovery from inactivation time constant (τ_recovery_) d (msec)
Wild type (A_WT_B_WT_)	11950±248	0.27±0.01	16460±836	43613±1420	2.65±0.10	−0.57±0.03	0	3728±108
T248A (A_WT_B_T248A_)	7957±255	0.20±0.02	9981±1336	39676±3416	3.97±0.40	−0.81±0.09	0.24±0.095	3353±254
Y415A (A_Y415A_B_WT_)	6093±137	0.22±0.01	7841±484	27321±1107	3.48±0.16	−0.73±0.04	0.16±0.05	5444±172
S428A (A_S428A_B_WT_)	9469±138	0.31±0.04	13811±2348	30117±2909	2.18±0.30	−0.46±0.11	−0.11±0.11	3082±48
T248A;Y415A (A_Y415A_B_T248A_)	2311±278	0.17±0.01	2801±354	13205±1483	4.71±0.27	−0.91±0.09	0.34±0.095	6434±669
T248A;S428A (A_S428A_B_T248A_)	2385±114	0.19±0.02	2937±429	12686±1233	4.32±0.47	−0.86±0.10	0.29±0.1	2580±84
Y415A;S428A (A_Y415A;S428A_B_WT_)	3950±65	0.26±0.03	5381±803	14849±1321	2.75±0.33	−0.60±0.10	0.03±0.1	3229±94
Y415A;S428A;T248A (A_Y415A;S428A_B_T248A_)	900±25	0.18±0.02	1101±161	4918±464	4.46±0.50	−0.88±0.10	0.31±0.1	9645±577

*K*_I_) and the free energy for inactivation gate closure (*ΔG*_inactivation_), for all single-, double- and triple-mutants used to calculate the coupling free energies during inactivation gating.^a^ The table displays the forward and backward time constants for slow inactivation gating (at 0 mV) along with the inactivation equilibrium constant (

*K*_I_ were calculated according to *K*_I_ = *k*_f_/*k*_r_ (see **Materials and Methods**).^b^ Values for

*ΔG*_inactivation_ were calculated according to *ΔG*_inactivation_ = −RTln *K*_I_.^c^ Values for

**Materials and Methods**).^d^ Values for the slow recovery phase are presented (see

*ΔΔG*_inactivation_ were calculated according to *ΔΔG*_inactivation_ = (−*RT*ln (*k*_f_/*k*_b_)_wt_ −−*RT*ln (*k*_f_/*k*_b_)_m_).^e^ Values for

The gating insensitivity of the T248, Y415 and S428 residues during inactivation gating implies that interactions across the upper interface that were shown to stabilize the open channel state are also preserved in the slow inactivated state. To formally assess this claim, we employed DMC analysis. We expected that coupling values (*Δ* ^2^ *G*_(*i,j*)_) close to zero would be found for the different upper interface residue pairs during open-to-inactivated gating transition (**O** ↔ **I**), since DMC coupling analysis evaluates the strength of interactions in one state relative to another [16]–[18]. In other words, we expect the effects on slow inactivation gating upon combining upper interface mutations to sum up in an additive manner. This indeed turned out to be the case. Progressively greater effects on the entry to and recovery from inactivation rates are observed as the number of mutations increases (**Table 4**, compare values for the single-, double- and triple-mutants). This is further reflected in energetic terms. Applying DMC analysis to the open-to-inactivated gating transition (*K*_I_, at 0 mV) (**Table 4**) revealed coupling values of −0.11 (±0.15), −0.21 (±0.18) and −0.02 (±0.16) kcal/mol for the three respective Y415;S428, T248;Y415 and T248;S428 upper interface residue pairs. Consistent with this result, the three-way coupling (*Δ* ^3^ *G*_(*i,j*)*k*_) calculated for the upper interface residue triad was found to be ∼0.05 (±0.20) kcal/mol. Taken together, the mutation-related gating insensitivity of the upper interface single-mutants during inactivation gating and the results of high-dimensional DMC coupling analysis in terms of magnitude and state-dependency suggest that the upper inter-domain inter-subunit interface does not undergo conformational rearrangements during channel slow inactivation gating.

## Discussion

**Figure 7:**
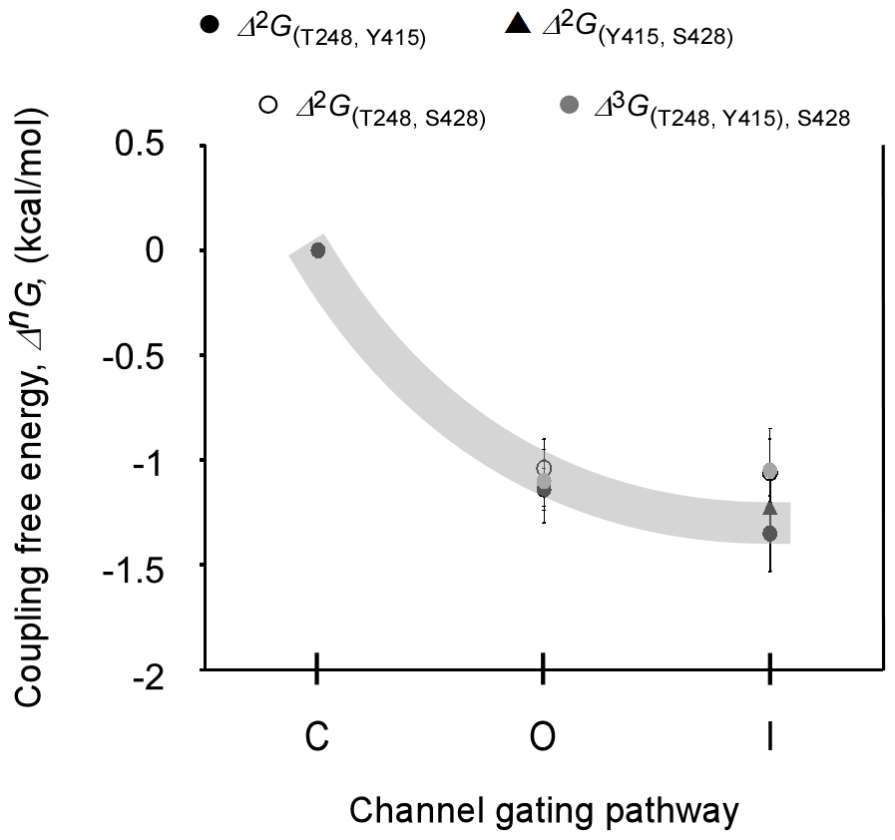
Changes in upper interface inter-subunit interactions along the reaction coordinate of channel gating. Changes in 2^nd^- or 3^rd^-order interaction strengths along the upper interface as a function of the reaction coordinate of Kv channel gating (**C** ↔ **O** ↔ **I**). Coupling free energies between the different core upper interface pairs during activation gate opening or inactivation gate closure were calculated by DMC analysis, normalized relative to the closed channel state. The coupling values indicated are at a reference voltage of 0 mV (see **Materials and Methods**). The gray stripe was schematically drawn to facilitate visualization of the state-dependency of the upper interface interactions during activation gating (**C** ↔ **O** transition).

**Figure 8:**
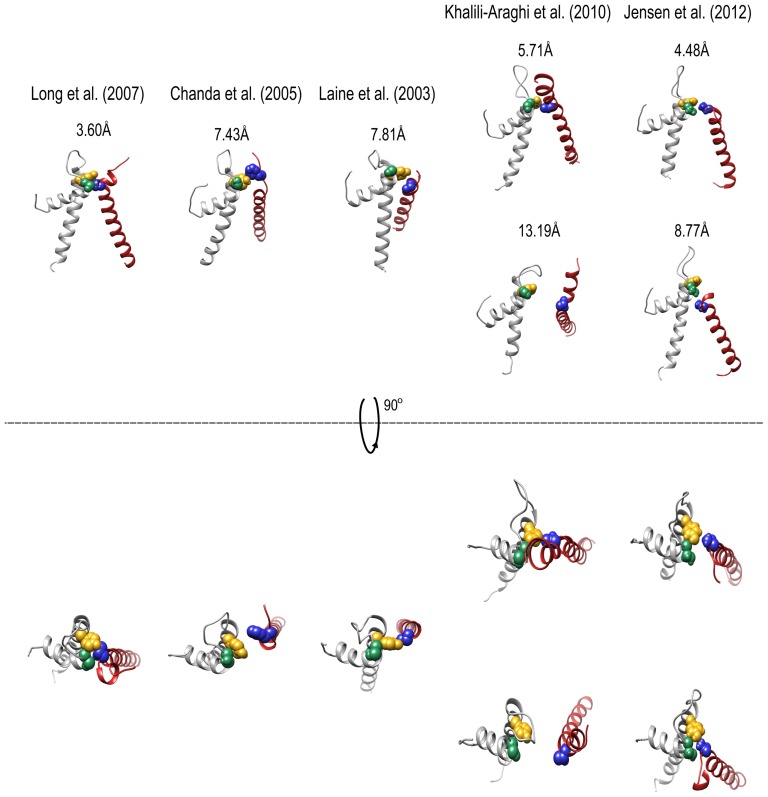
The intimate upper interface residue network loosens upon Kv channel closure. Side and top view comparisons of the upper interface residue network between the open Kv channel structure (left) and the different models of the closed channel state (indicated by the reference notation above each rectangle). Structures were first aligned using the sPDB viewer to allow a common coordinate system. Panel figures were prepared using the Molecular graphics UCSF Chimera package [34]. The number in each panel represents the shortest atom distance (in Å) between the T248 and Y415 residue pair. Note that in two closed state models ([29], [33]), apparent asymmetry is observed along the upper domain interface. Space-filling representations of the Kv S1 T248, S5 Y415 and P S428 residues are highlighted in blue, gold and green, respectively, as in **Fig 1C**.

In the present study, we evaluated the contributions of putative interactions across the upper interface of the Kv channel (**Fig. 1**) [8] to primary conformational transitions associated with channel gates, namely activation gate opening and (slow) inactivation gate closing, as delineated by the simple reductionist channel gating sequence: **C** ↔ **O** ↔ **I**. Our results indicate that moderate structural rearrangements along the upper domain interface occur during activation gating but not during inactivation gating. First, all three primary T248, Y415 and S428 upper interface residues forming the core of the intimate upper interaction surface ([8] **Fig. 1C**) are gating-sensitive and stabilize the closed channel state upon mutation (**Fig. 3**). These residues are gating-insensitive during the subsequent inactivation gating transition (**Fig. 6**). Second, double-mutant cycle coupling analysis of the different upper interface residue pairs revealed the existence of moderate coupling among the upper interface residues and the state-dependency of these interactions, only during activation gating (**Figs. 4** **–** **5**). This is realized by plotting the strength of the three primary upper interface interactions considered here (*Δ* ^2^ *G*_(*i,j*)_) as a function of the reaction coordinate of Kv channel gating [28] (**Fig. 7**). It can be seen that any of the three residue pairs considered are more strongly coupled in the open channel state than in the closed state by ∼1 kcal/mol. At the same time, these residues are similarly coupled during slow inactivation gating. The same coupling pattern is revealed for *Δ* ^3^ *G*_(*i,j*)*k*_, indicating that mutual coupling among the three upper interface residues prevails primarily in the open and inactivated channel states but not in the closed state, reflecting the occurrence of conformational rearrangements across the upper domain interface only during activation gating. Comparison of the open Kv1.2 channel structure with computed models of the closed Kv channel state [29]-[33], moreover, reveals differences across the upper interface, in support of the above conclusion (**Fig. 8**). As can be seen, the intimate interaction network among the upper interface residues observed in the Kv 1.2 structure is loosened upon channel closing. Indeed, the closest interaction distance between the T248 and Y415 residues is found in the open channel structure. Interestingly, recent structural data on members of the closely related voltage-gated sodium channel family, solved in different conformations, revealed structural changes along the upper interface [35]–[37].

**Figure 9:**
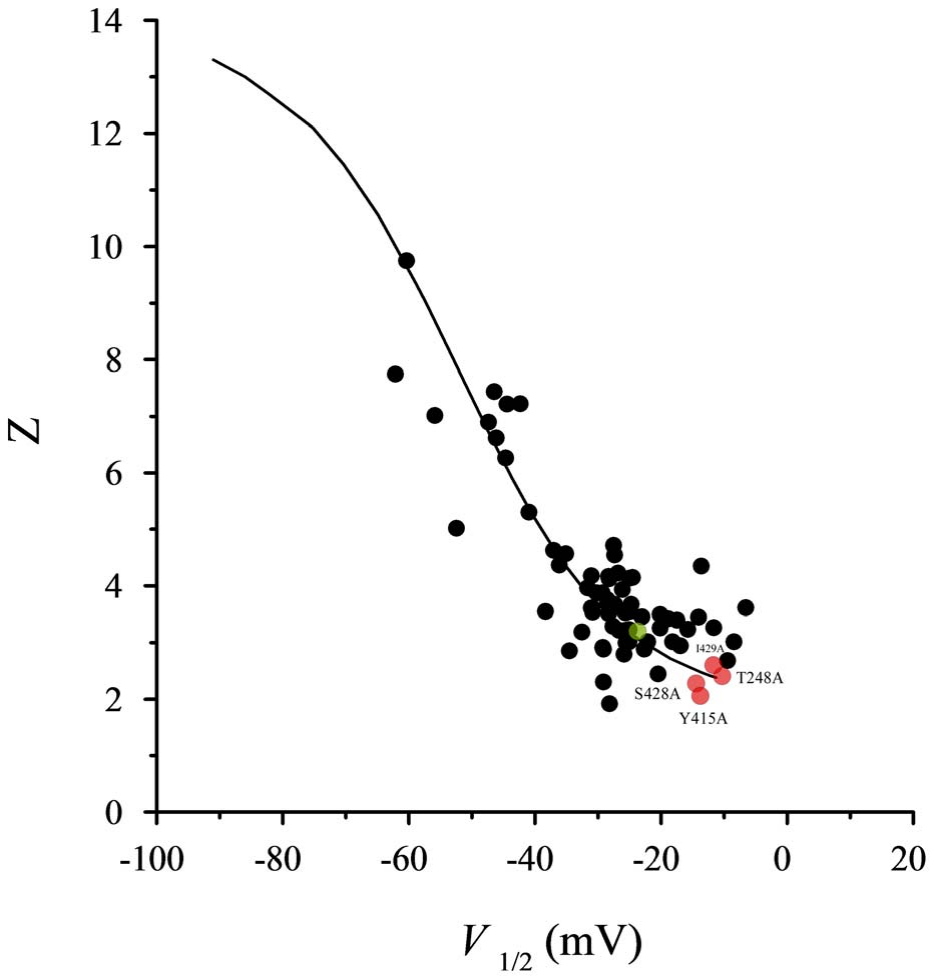
The upper interface single-mutant gating parameters conform to the expected trend for perturbations that primarily affect late concerted pore opening. The Z and V_1/2_ phenomenological gating parameter values of the T248A, Y415A, S428 and I429A upper interface single-mutants mapped onto the Z-V_1/2_ phase space (red circles), in the context of a previously observed experimental correlation between the Z and V_1/2_ values of pore mutant *Shaker* channel proteins (black circles). The green point is for the wild type *Shaker* channel values. The smooth curve corresponds to the expected theoretical trend for the 16-state gating model of the *Shaker* channel (Zagotta, Hoshi and Aldrich (1994), *J Gen Physiol* 103: 321-362), where perturbations are assumed to only affect late concerted pore opening (*L*) transition. Refer to reference [11] for further details.

Taken together, our results support a scenario whereby the upper inter-subunit interaction surface serves as a focus for mutual structural rearrangements between the pore and voltage-sensor domains during the late pore opening transition. Several lines of evidence support this conclusion. First, the Y415, S428 and I429 residues, shown here to interact with the S1 T248 residue across the inter-subunit domain interface, are also part of the allosteric communication trajectory along the pore domain important for late concerted pore opening [11], [17]. Second, the compatibility between the open channel structure [8] and the gating effects seen upon mutation, in particular the fact that mutations stabilize the closed channel states (**Fig. 3**) and that measured interactions are stronger in the open channel state (**Figs**. **4** **–** **5** and **Table 3**), argue that we evaluated direct interactions that contribute to late pore opening. Third, the fact that all upper interface single-mutant channels exhibit reduced Hill coefficients of channel gating (as indicated by the slope factor; **Table 2**) [38] further indicates that cooperativity in activation gate opening, as brought about by inter-subunit interactions, is compromised upon mutation. Furthermore, as can be seen in **Fig. 9**, the values for the upper interface single-mutants gating parameters (activation midpoint and slope) well fit the experimental and theoretical relations between Z and V_1/2_ expected for perturbations that primarily affect the late concerted pore opening transition [11]. Fourth, it has been shown that following resting-to-activated transition of the *Shaker* Kv channel voltage-sensors, the paddle-sensor motif and the pore domain undergo structural rearrangements focused around the upper interaction surface during the final late pore opening transition [39]. Specifically, it was shown that during late channel opening transition, the arginine at the R1 position of the paddle motif (R362 in the *Shaker* channel) forms an intimate interaction with the F416 pore residue (adjacent to the Y415 interface residue studied here) [39]. Finally, an elaborate study of the Kv 7.1 channel revealed a key role for the extracellular part of the S1 segment, a region that contributes to the voltage-sensor side of the upper domain interface in steering late S4 motions during activation gate opening [40].

Combining these findings, we suggest that late conformational rearrangements between the voltage-sensing and pore domains brought about by the upper inter-subunit domain interaction surface contribute to the concerted nature of the late pore opening transition of the Kv channel. However, considering the magnitude of upper interface interactions evaluated here (∼1 kcal/mol) and the comparison between the open structure and the closed channel state models, such rearrangements are not dramatic or extensive, as compared to those occurring at the lower interface [8], [29]–[33]. Whether rearrangements also occur along the upper interface during early voltage sensor transitions still remains unknown. Thus, the assertion for a stiff upper domain interaction surface during voltage sensor transitions that may serve to efficiently transmit the torque generated by the movements of the paddle elements to the activation gate of the pore, as previously suggested [9], [14], requires further validation.

From the broad allosteric perspective, it is interesting to note that while our previous studies on allosteric communication between the Kv channel pore gates revealed that inter-subunit interactions, in particular at the lower domain interface where the activation gate resides, stabilized the closed conformation of the channel (reflected in an interaction defined by a ‘+’ coupling sign) [11], [17], in the case of the upper interface, other inter-subunit interactions stabilize the open channel state (reflected in an interaction defined by a ‘−’ coupling sign). This difference in inter-subunit interaction patterns upon Kv channel activation gate opening illustrates Perutz's general notion that during the major conformational transition of an allosteric protein, when inter-subunit interactions that stabilize one conformation are broken, other interactions stabilizing the second conformation are formed concomitantly [12].

## Supporting Information

Figure S1 **Interfering with the native context of the pivotal T248 affects gating and stabilizes the closed channel state.** **A**) Multiple sequence alignment of several members of the Kv channel family and of two members of the Nav channel family reveals the conservation of the S1–S2 loop region, in particular around the pivot T248 residue. The alignment was generated using the STRAP program ‘STRAP’ (Gille C, Frömmel C (2011): editor for STRuctural Alignments of Proteins. *Bioinformatics*, **17**:377–378). **B**) Sequence and annotation of mutations designed to perturb the native structural context of the pivotal T248 residue. Native, non-mutated positions are indicated in bold (**C**) Voltage-activation data for the functional K^+^ channel proteins indicated in (**B**). Smooth curves correspond to a two-state Boltzmann function. For clarity, no error bars are plotted for the different data points of the different curves. These values, however, are in a range of 5% of the reported values.
(EPS)

Table S1 **Distances between upper interface residue pairs in the Kv 1.2 open channel state.** ^a^Distances along the Kv 1.2 paddle chimera structure (PDB code: 2R9R) were measured using the SPDB viewer program. ^b^The primary residues that form a network of hydrogen bonding interactions addressed in the current study are highlighted in red.
(DOC)
