# Supplementary material for: Inter-Subunit Interactions across the Upper Voltage Sensing-Pore Domain Interface Contribute to the Concerted Pore Opening Transition of Kv Channels
Source: PLoS One. 2013 Dec 10;8(12):e82253. doi: 10.1371/journal.pone.0082253 (PMC3858418; doi:10.1371/journal.pone.0082253)
Supplement: Table S1 — Distances between upper interface residue pairs in the Kv 1.2 open channel state. aDistances along the Kv 1.2 paddle chimera structure (PDB code: 2R9R) were measured using the SPDB viewer program. bThe primary residues that form a network of hydrogen bonding interactions addressed in the current study are highlighted in red. (DOC) [file pone.0082253.s002.doc]

**Supplementary Information Table**

**Table S1: Distances between upper interface residue pairs in the Kv 1.2 open channel state**

| Distance [Å]^a^ | Interacting atom | Pore domain residue partner | Voltage-sensor domain S1 residue |
| --- | --- | --- | --- |
| 3.88 | Cγ2-Cδ1 | S5: V408 (I336) | I241 (I177) |
| 3.79 | CZ-Cε2 | S5: F416 (F344) | F244 (F180) |
| 3.6 | Cγ2-Cε2 | **S5: Y415** (Y343) | **T248** (T184)^b^ |
| 3.75 | Cβ-CZ |  |  |
| 3.88 | Cβ-OH |  |  |
| 3.6 | O-Cα | **P: S428** (S356) |  |
| 3.76 | Oγ1-Cα |  |  |
| 3.22 | O-Cβ |  |  |
| 2.93 | Oγ1-NH | **P: I429** (I357) |  |
| 3.45 | Oγ1-Cδ | P: P430 (P358) |  |
| 3.29 | Nε-OH | S5:Y415 (Y343) | K253 (R189) |
